# A re-evaluation of Stuart's police officer stigma scale: Measuring mental health stigma in first responders

**DOI:** 10.3389/fpubh.2022.951347

**Published:** 2022-09-20

**Authors:** Zachery Burzee, Clint Bowers, Deborah Beidel

**Affiliations:** ^1^Department of Psychology, University of Central Florida, Orlando, FL, United States; ^2^UCF RESTORES, University of Central Florida, Orlando, FL, United States

**Keywords:** stigma, mental health stigma, first responders, police, firefighters, self-stigma, public stigma

## Abstract

Stigma about mental illness is often identified as one of the most prominent obstacles to seeking mental health services. This seems to be particularly true among first responders. Unfortunately, the research regarding stigma in first responders is lacking. This may be due, in part, to the absence of appropriate measurement tools to allow such research. Police Officer Stigma Scale (POSS) has recently been developed to address this issue, but its psychometric properties have gone largely untested. Therefore, this study sought to identify the underlying factor structure and internal consistency of the POSS. This paper used a sample of 135 first responders. Using factor analysis with an orthogonal rotation on Stuart's 11-item POSS, the participant's results revealed two main components, accounting for a total of 72.79% of the overall variance. Factor one is “maltreatment of colleagues with a mental disorder,” and is associated with six of the 11 items on the scale, such as “Most police officers believe that a colleague who has had a mental illness is not trustworthy.” Factor two is “fear of disclosing a mental disorder.” It includes items such as “Most police officers would not disclose to a supervisor/manager if they were experiencing a mental illness.” Findings from this research are similar to the results of previous studies with components such as unwillingness to disclose a mental health condition, fear of how the public will treat an individual with a mental disorder, and anger toward those who decide to seek treatment or get diagnosed with a mental illness. These findings imply that Stuart's POSS is reliable but needs to include two components rather than one. With the two main components, further research can now be conducted to understand why and ultimately mitigate maltreatment or stigma against first responders with a mental health condition.

## Introduction

First responders [police, firefighters, emergency medical technicians (EMTs), and military members] perform their duties in extremely stressful circumstances. These stressors include heat, long hours, intense workload (1), and even threat of personal harm or death (2). Therefore, it is not surprising that these employees are at risk for a host of negative outcomes such as depression (3), substance abuse (4), post-traumatic stress disorder (5), and suicide (6).

Although there has been an increase in mental health resources for first responders, many continue not to receive needed care (7). One of the most frequently cited explanations for this problem is stigma toward mental illness. Many first responders fear that they may experience negative career consequences (8). Additionally, there is often a personal set of negative attitudes toward mental illness that might threaten their self-esteem. Since these attitudes often exist in a social climate that values strength and devalues weakness, their effects on help-seeking might be particularly pronounced (9).

The assertion that stigma toward mental illness is prevalent among first responders, and related to the under-utilization of mental health service, is based largely on anecdotal evidence and qualitative data [cf. (10)]. However, the available data do seem compelling. For example, a large survey study by Drew and Martin (11) found that more than 90% of respondents believed that stigma was related to a lack of help-seeking behavior. A meta- analysis by Haugen et al. indicated that an average of 33% of first responders reports some type of stigma belief. In fact, the connection between stigma and help-seeking in this population is so well-accepted that many programs have been created to reduce stigma. Peer support, psychoeducation, and other awareness programs have been especially popular in this regard (12).

Unfortunately, the quantitative research base has not evolved quickly enough to provide substantial guidance in this area. Although there has been a groundswell of interest in stigma toward mental illness, the research is in its relative infancy and there is yet to be a convergence of thinking regarding the theoretical aspects of the phenomena. However, it's generally accepted that stigma toward mental illness is a multi-dimensional construct. Generally speaking, this includes self-focused and an other-focused components (13).

Measurement in this area, consequently, is also emerging. However, as described by Fox et al. (13), many of these measures were developed for one particular study and have not provided sufficient psychometric data to be viewed as valid or generalizable. Furthermore, the proliferation of single-use scales makes it difficult to synthesize results across studies. For example, the current scales include factor structures ranging from one to as many as six. The authors conclude that there is a need not for more scales, but more psychometric research on exiting scales. The present study seeks to respond to that call.

Furthermore, the existing scales were generally developed for broad research into the construct of stigma but were not well-tailored to the specific issue of stigma in the workplace (14). To respond to this problem, Szeto et al. developed the Open Minds Scale for Workplace Attitudes [OMS-WA; (15)]. A series of studies have converged to indicate that this measure might be more appropriate for workplace studies than previous, broader, measures [see Szeto et al. (15) for a review]. They argue that this more precise information is particularly helpful when used to develop interventions to reduce workplace stigma.

Building on this success, researchers suggest that additional benefit might be gained from further tailoring stigma measures to specific, high-risk workplaces (15). For example, this approach has been used effectively to understand stigma issues among healthcare providers (16, 17). This has allowed this profession to develop targeted interventions such as additional training for medical students (16).

Similarly, Stuart (18) developed the *Police Officer Stigma Scale (POSS)* to assess mental health stigma issues in that group of professionals. Stuart based the POSS on Link's (19) Perceived Devaluation and Discrimination Scale (PDDS). The PDSS seeks to assess stigma by asking the participant's perceptions about their peer's beliefs about mental illness. It was believed that this approach might lead to more honest reporting than asking them about their own beliefs. The PDDS has been used in a variety of settings. Studies of its psychometric properties have suggested that it has two underlying factors (20, 21). These factors have been characterized as “perceived acceptance” and “perceived discrimination” (20).

The POSS attempted to translate the PDDS into a scale that is optimal for police officers. The POSS uses five necessary themes for police, including acceptance by others, perceived trustworthiness, employment discrimination, taking opinions less seriously, and treatment as a sign of personal failure. The POSS includes six more themes because they relate to police culture, including disclosing to a colleague, announcing to a supervisor/manager, avoiding seeking help, expectations of discrimination at work in promotions, general expectations of discrimination, and not wanting a supervisor with a mental illness. Item-rest correlations for a single factor solution were all above 0.4, indicating good inter-correlations. The POSS reports a high Cronbach's alpha (α = 0.82), implying good reliability (18). However, Stuart (18) did not obtain the two-factor structure from the original PDDS. Rather, she reports that a one-factor solution better fit the data. However, the manuscript does not provide the data required to fully evaluate the underlying factor structure. For that reason, we sought to re-evaluate the POSS factor structure with a different sample. For our sample, we used police, firefighters, and dispatchers to derive sufficient power for a factor analysis. A recent paper by Bowers et al. reported no difference in stigma between these three groups in a different sample of first responders (22).

## Methods

### Participants

Participants for this study were 135 first responders that attended a mandatory Mental Health Awareness training session in central Florida. The sessions were delivered throughout the state of Florida in the Fall of 2021. Participants volunteered to participate without renumeration. After consenting, participants completed the measure online before attending the training session. Sixty participants were police officers, 48 were firefighters/EMTs, three were dispatchers. Demographic data about the sample are provided in Table 1.

**Table 1:** Demographic characteristics table.

**Characteristic**	**Guided self-help**
	***n***	**%**
**Gender**		
Female	14	13
Male	94	87
**Marital status**		
Single	26	24
Married/partnered	72	67
Divorced/widowed	9	8
**Highest educational level**		
Middle school	0	0
High school/some college	4	2.5
University or postgraduate degree	103	97.5

Participants were on average 39.5 years old (SD = 10.1).

Data were collected in accordance with the ethical standards of the American Psychological Association. The study was evaluated and approved by the university's Institutional Review Board.

### Measures

Stigma toward mental illness was assessed using the Police Officer Stigma Scale POSS, Stuart (18). The POSS is an 11-item scale designed to measure mental health stigma among police officers. Rather than assessing the participant's perception of the general public, the POSS targets beliefs held by fellow officers. The scale was adapted for use with firefighters and dispatchers for the current study (i.e., “Firefighter” was used instead of officer). Participants respond using a 5-point Likert Scale with anchors ranging from “Strongly Agree to Strongly Disagree.” The POSS is typically scored by simply summing the responses. However, we used individual item responses for the following analyses. The items are presented in Table 2.

**Table 2:** Rotated factor loadings by item.

**POSS rotated item component matrix**
**Item**	**Factor 1 “Maltreatment of colleagues with a mental disorder”**	**Factor 2 “Fear of disclosing a mental disorder”**
Most police officers would not disclose to a supervisor/manager if they were experiencing a mental illness.	0.138	0.884
Most police officers would not disclose to a colleague if they were experiencing a mental illness.	0.181	0.823
Most police officers would expect to be discriminated against at work if they disclosed that they were experiencing a mental illness.	0.495	0.667
Most police officers would not want a supervisor/manager who had a mental illness.	0.317	0.733
Most police officers think that being treated for a mental illness is a sign of personal failure.	0.552	0.628
Most police supervisors/managers would not consider an application for promotion from an officer who has had a mental illness.	0.66	0.476
Most police officers would not seek professional help if they were experiencing a mental illness.	0.268	0.789
Most officers would not willingly accept a colleague with a mental illness as a partner.	0.735	0.343
Most police officers would think less of a colleague who has had a mental illness.	0.867	0.301
Once they know a colleague has had a mental illness, most police officers would take their opinions less seriously.	0.857	0.246
Most police officers believe that a colleague who has had a mental illness is not trustworthy.	0.899	0.096

### Procedure

Participants completed a pre-test online before attending the session. One hundred and thirty-five participants participated in the training. One hundred and eleven completed at least a portion of the pre-test assessment. The volunteers were debriefed after the training session was complete.

## Results

Internal consistency was evaluated for all items in the scale. The analysis yielded an alpha estimate of 0.84. A principal-components factor analysis was then conducted on the 11 items. One hundred and thirty-five first responder sample. This sample size is deemed sufficient for an 11-item scale (23).

**Figure 1:**
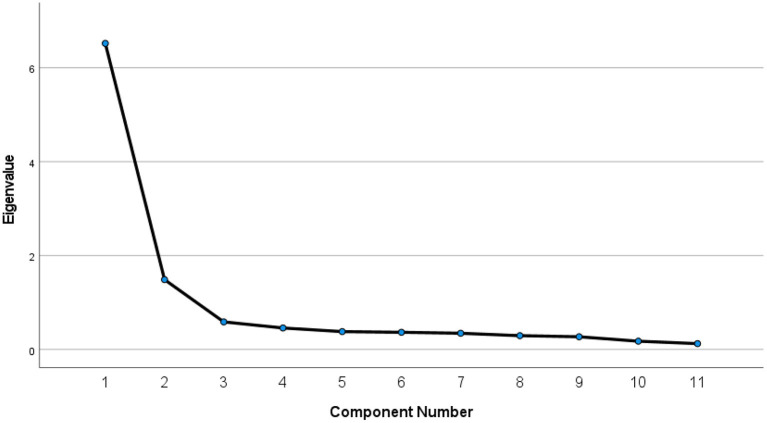
Scree plot for entire sample.

**Figure 2:**
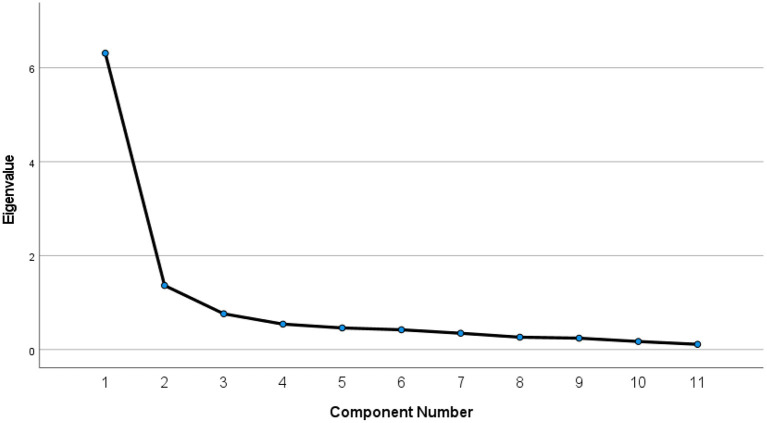
Scree plot for police only.

Examining the initial eigenvalues (before rotation), one could conclude that two components were extracted from the data. Component one had an eigenvalue of 6.521 and explained 59.278% of the variance. Component two had an eigenvalue of 1.487 and explained 13.516% of the variance with a combined total of 72.794%. Component three gave an eigenvalue of 0.586. According to Field (24), Kaiser's criterion of retaining factors is to discard factors with eigenvalues under one and keep factors with eigenvalues >1. In short, two components were included, and nine were discarded. This factor solution is illustrated in the Scree plot in Figure 1. Interestingly, an identical factor analysis using only the police officers in this sample yielded a very similar two-factor solution. This scree plot is provided in Figure 2.

The modifications made to create the POSS make it impossible to compare the factor loadings with the two-factor solution of the PDDS. However, when one looks at the factor loadings there are clear distinctions between factors one and two. Questions one through five and seven are explained by component two, while questions six, and eight through 11 are explained by factor one. Examining the individual item loadings, factor one seems to best be described as items dealing with perceptions of, while the items that load on Factor two appear to *how others with mental illness are treated* relate to concerns about *disclosing a mental disorder*. This aligns closely with the factor loadings of the original PDDS and the summary model developed by Fox et al. (13).

## Discussion

Mental health stigma is considered to be a primary barrier to care among first responders (25). For that reason, there has been a surge of effort to confront and correct stigmatizing beliefs among these workers. Foundational to this effort, however, is the ability to assess stigma at these workplaces to understand the nature of the stigma within the organization in order to create optimal interventions. As noted earlier, there is no shortage of available measures of stigma toward mental illness. At this point, the best route for researchers might be to explore the psychometric properties of these measures in hope of identifying sound measures for use in practice (13).

As discussed above, stigma has frequently been conceptualized as a multi-dimensional construct (25, 26). Broadly speaking this can be conceptualized as attitudes toward mental illness in others, and attitudes toward mental illness in oneself. These two different sub-types might have different impacts on the organization. Attitudes toward others might influence team performance, trust, and group self-efficacy (27). Additionally, they may influence the quality of one's work when dealing with mentally ill people (28). Conversely, attitudes toward illness in oneself may influence the decision to admit symptoms and seek treatment. Most anti-stigma programs have focused on the latter factor (29), so there might be considerable benefit to addressing the issue of attitudes toward others.

Stuart's (18) POSS is, to the best of our knowledge, the only measure created to assess stigma specifically within first responders. In a first test of this scale, Stuart reported that the measure was best described by a single-factor solution. However, this result is contrary to the current theories of stigma and also to the factor structure of the PDDS, on which the POSS is based. For those reasons we sought to replicate the Stuart study using a different sample of first responders. Our results support a two-factor solution, with one factor apparently focused on perceived maltreatment of others with mental illness, and a second factor related to concerns about disclosing a mental illness.

The two-factor solution aligns well with theoretical models of mental health stigma as a multi-faceted construct. For example, our obtained solution seems to match Haugen et al.'s notion of stigma awareness and stereotype avoidance and Fox et al.'s (13) summary model of the literature. This is important because the validity of the measure is dependent upon the measure's ability to assess the totality of the construct. A one-factor measure does not represent most of the current theories of stigma and may limit our ability to inform interventions optimally. Specifically, it may not reveal whether interventions should be targeted at the individual, the organization, or both. Interestingly, it should be noted that the Open Minds Scale for Healthcare providers yielded a very similar factor structure, lending credence to an underlying two-factor conceptualization of stigma toward the mentally ill (30). However, a variety of scales have been developed for this population and there is not an agreement on the underlying structure [see (31) for a review].

### Limitations and future research

It should be noted, however, that the present study is different from the Stuart study in a few ways that might be significant. First, the current results are based on a sample of first responders from one U.S. state while the Stuart study was conducted with Canadian officers. It seems likely that there are cultural differences in mental health stigma (32). Second, the stated goal of the Stuart study was to find a “a simple factor structure where all items loaded on a single factor” (18), while our goal was to find the optimal structure to fit the data. Finally, the present study was based on a sample that included police, firefighters, and dispatchers while Stuart used only police. It is noted, however, that we obtained the same result when using only the police officers in our sample.

The accurate assessment of mental health stigma is a precursor to the development of effective interventions (13, 25). Our goal in this study was to determine whether mental health stigma among first responders is better assessed as a multi-factor construct. These results suggest that there may be an advantage of using two factors to interpret results of the POSS. Future research should investigate this assertion with a larger, broader sample to replicate these results. Furthermore, a confirmatory factor analysis with a larger, broader, sample is likely to shed even more light on the underlying dimensions of this critical construct.

## Conclusion

The proliferation of assessment tools to measure stigma toward mental illness offer tremendous promise for an enhanced understanding of the important concept. However, there is a need to examine the psychometric properties of these scale to ensure their optimum use. The present paper seeks to respond to this challenge by re-examining some of the psychometric properties of the POSS (18). We conclude that this measure might be better used as a two-factor assessment than a single-factor one. In doing so we may provide more detailed guidance to organizations trying to combat mental health stigma.

## Data availability statement

The original contributions presented in the study are included in the article/supplementary material, further inquiries can be directed to the corresponding author.

## Ethics statement

The studies involving human participants were reviewed and approved by University of Central Florida. The patients/participants provided their written informed consent to participate in this study.

## Author contributions

All authors listed have made a substantial, direct, and intellectual contribution to the work and approved it for publication.

## Conflict of interest

The authors declare that the research was conducted in the absence of any commercial or financial relationships that could be construed as a potential conflict of interest.

## Publisher's note

All claims expressed in this article are solely those of the authors and do not necessarily represent those of their affiliated organizations, or those of the publisher, the editors and the reviewers. Any product that may be evaluated in this article, or claim that may be made by its manufacturer, is not guaranteed or endorsed by the publisher.
